# Treatment-related pain in refractory cancer pain: prevalence, mechanisms, and clinical implications in a tertiary referral cohort

**DOI:** 10.1007/s00520-026-10886-6

**Published:** 2026-06-12

**Authors:** Rotem Agur, Barak Cohen, Mulham Sabbah, Silviu Brill, Maor Staierman, Dorin Goldsmith, Morsi Khashan, Uri Hochberg

**Affiliations:** 1 Division of Anesthesiology, Tel Aviv Sourasky Medical Center, Tel Aviv, Israel; 2 Gray Faculty of Medical and Health Sciences, Tel Aviv University, Tel Aviv, Israel; 3 Spine Surgery Unit, Neurosurgical Department, Tel Aviv Sourasky Medical Center, Tel Aviv, Israel; 4 Institute of Pain Medicine, Division of Anesthesiology, Tel Aviv Sourasky Medical Center, Tel Aviv, Israel

**Keywords:** Cancer survivorship, Treatment-related pain, Refractory cancer pain, Neuropathic pain, Multidisciplinary pain management

## Abstract

**Background:**

The long-term effects of cancer treatment are increasingly driving persistent pain in cancer survivors; however, the contribution and clinical profile of cancer treatment–related pain (CTRP) in refractory pain populations remain poorly defined.

**Methods:**

We conducted a retrospective cohort study of consecutive patients (*N* = 622) referred to a tertiary multidisciplinary cancer pain clinic for initial evaluation of refractory pain (January 2021-June 2025). Pain etiology was classified using ICD-11 criteria as treatment-related pain (TRP; MG30.11), tumor-related pain (MG30.10), or non–cancer-related pain. The multivariate logistic regression analysis identified factors associated with TRP attribution.

**Results:**

TRP was identified in 42.1% of patients (*n* = 262). Patients with TRP were younger than those with tumor-related pain (mean 58.1 ± 14.8 vs 64.2 ± 12.8 years, *p* < 0.001). Peripheral neuropathy was strongly associated with TRP attribution (adjusted odds ratio 9.18; 95% CI 2.56–32.83). Neuropathic (46.2%) and mixed (35.9%) mechanisms predominated in TRP, whereas nociceptive mechanisms were more common in tumor-related pain.

**Conclusions:**

Treatment-related pain constitutes a major component of refractory cancer pain and demonstrates a distinct clinical and mechanistic profiles. Its predominance among younger survivors supports its characterization as a survivorship-associated morbidity rather than an end-of-life phenomenon. These findings support the earlier recognition and integration of mechanism-informed, multidisciplinary supportive care strategies to improve outcomes in patients with persistent treatment-related pain.

**Supplementary Information:**

The online version contains supplementary material available at 10.1007/s00520-026-10886-6.

## Introduction

Cancer-related pain is common and often disabling, affecting quality of life, function, and psychological well-being. Epidemiological data indicate that 30–55% of patients experience clinically significant pain during active treatment, with prevalence exceeding 60% in advanced disease and remaining a substantial burden throughout survivorship [1–4]. Historically, clinical attention has focused on pain arising from tumor burden. As survival has improved, the pain landscape has expanded to include cancer treatment–related pain (TRP), an unintended consequence of modern cancer therapies.

In ICD-11 [5, 6], chronic pain attributed to active cancer is classified separately from chronic pain attributed to cancer treatment—chronic cancer-related pain (MG30.10) and chronic post-cancer treatment pain (MG30.11). Unlike the primarily inflammatory or mechanical nociception typical of tumor growth, TRP often presents as a mixed pain phenotype [7], involving concurrent nociceptive processes—such as radiation-induced fibrosis or hormone-related arthralgia—and neuropathic injury, including chemotherapy-induced peripheral neuropathy (CIPN) or post-surgical nerve damage [8–10].

Immune checkpoint inhibitors (ICIs) further broaden this spectrum, introducing syndromes characterized by severe musculoskeletal inflammation alongside neurologic features [11–14]. Even when clinically significant, TRP may be under-recognized or inconsistently documented, leading to misattribution to disease progression rather than treatment-related effects. This misclassification may result in ineffective escalation of conventional analgesics and contribute to persistent symptoms and increased risk of chronic pain.

Patients with cancer whose pain remains inadequately controlled despite optimized, guideline-supported pharmacologic management are often considered to have refractory cancer-related pain [15–17]. These patients represent a particularly challenging clinical population, with substantial symptom burden, impaired quality of life, and complex needs that strain oncology, palliative, and supportive care services. In this context, TRP may be under-recognized in routine oncology practice, and barriers to systematic pain assessment and timely, mechanism-directed management may contribute to persistent pain and functional limitation [18, 19]. Although approximately 10–20% of patients experience refractory pain, systematic data on the contribution of treatment-related factors in this population remain limited. Existing studies typically focus on individual syndromes, and TRP has not been comprehensively characterized in specialized referral settings.

Despite increasing recognition of cancer survivorship as a distinct clinical phase, the contribution of treatment-related pain among patients with refractory cancer pain remains insufficiently defined. In tertiary referral settings, where patients present with complex and persistent symptoms despite guideline-based management, understanding the relative contribution and clinical profile of TRP has direct implications for supportive care strategies.

In our tertiary, university-affiliated multidisciplinary center, we established a specialized clinic for patients with refractory cancer-related pain following inadequate response to standard pharmacologic management. This referral population allows direct assessment of the relative contribution of tumor-related versus treatment-related pain in a tertiary refractory setting.

The objective of this study was to quantify the prevalence and define the clinical and mechanistic profile of cancer treatment–related pain (TRP) relative to tumor-related pain in a tertiary cohort of patients referred for refractory pain.

## Methods

### Study design, setting, and oversight

This retrospective cohort study used data from a specialized Cancer-Related Pain Clinic at a tertiary, university-affiliated center. Patients referred for first evaluation of refractory cancer-related pain between January 2021, and June 2025 were included. The clinic operates within a multidisciplinary framework, including medical, surgical, and interventional pain specialists. The institutional ethics committee approved the study (0414–25-TLV).

Patients were referred following inadequate response to guideline-based pharmacologic management, typically consistent with escalation along the WHO analgesic ladder. Accordingly, the study population represents a clinically complex cohort with persistent pain despite prior standard treatment, often associated with substantial symptom burden.

### Data abstraction and quality control

Six pain physicians extracted demographic, oncologic, and pain-related variables using predefined coding fields and standardized operational definitions. Pain etiology was assigned using a structured classification process incorporating clinical history, temporal relationship to cancer treatment, anatomic distribution, and compatibility with recognized pain syndromes. Each case was categorized as cancer treatment–related pain (TRP), tumor-related pain, or non–cancer-related pain.

A seventh physician performed secondary quality control, reviewing all cases and resolving discrepancies through consensus and repeat chart review.

### Pain etiology groups and assignment

Etiology was classified into three mutually exclusive ICD-11 categories: (1) cancer treatment–related pain (TRP; MG30.11), (2) tumor-related pain (MG30.10), and (3) non–cancer-related pain. Pre-existing chronic conditions predating cancer were categorized as non–cancer-related.

TRP was identified using a structured framework based on compatible syndrome patterns and temporal association with cancer-directed therapy, as documented in clinical records. This included systemic therapy–related syndromes (e.g., chemotherapy-induced peripheral neuropathy), persistent post-surgical pain in the operative field, and radiotherapy-associated syndromes (e.g., plexopathy) with a corresponding anatomic distribution. Cases with ambiguous documentation were conservatively classified as tumor-related or non–cancer-related, and those with insufficient data were excluded.

For analytic purposes, each patient was assigned a single primary pain etiology, although overlapping mechanisms may coexist clinically.

### Pain diagnosis, mechanism, and clinical variables

Pain mechanisms were classified as nociceptive, neuropathic (per the updated grading system [20]), or mixed (co-existing mechanisms at the same site). Pain diagnoses were categorized into 14 predefined groups (e.g., post-surgical pain, bone metastasis).

Additional abstracted variables included demographics, primary cancer type, disease status (active vs. non-active), and cancer-directed treatment exposures.

Pain duration was categorized as > 6 months versus ≤ 6 months to assess symptom persistence in this refractory cohort. Although ICD-11 defines chronic pain as ≥ 3 months, we applied a more stringent 6-month threshold to capture more persistent and clinically entrenched pain in a tertiary referral population.

Management strategies, including medical cannabis use and interventional procedures, were recorded. Detailed operational definitions are provided in the Appendix.

### Vital status and time-to-death interval

Survival variables were summarized descriptively. Analysis included patients with confirmed vital status at data cutoff. For those who were deceased, the interval from index clinic visit to death was recorded as < 6, 6–12, or > 12 months. Differences between the groups (vital status and death-interval distributions) were evaluated using chi-square tests, which were also used to assess potential attrition bias across etiology groups.

### Statistical analysis

Continuous variables are reported as mean ± SD; categorical variables as n (%). Group comparisons utilized one-way ANOVA and chi-square tests. A multivariable logistic regression identified factors associated with TRP attribution, comparing TRP (treatment-related pain) vs tumor-related pain (cancer-related non-treatment pain only). To minimize incorporation bias, the primary model excluded treatment-exposure variables (surgery, radiotherapy, systemic therapy), which were instead analyzed in a secondary sensitivity model. Covariates included age, sex, cancer type, duration, mechanism (reference: mixed), and peripheral neuropathy. Analyses were performed using IBM SPSS Statistics (*p* < 0.05).

## Results

### Patient characteristics

A total of 622 patients met the inclusion criteria and were categorized by pain etiology: 262 (42.1%) with Treatment-Related Pain (TRP), 194 (31.2%) with cancer-related non-treatment pain (tumor-related), and 166 (26.7%) with non–cancer-related pain, representing the largest etiologic subgroup. Patients in the TRP cohort were significantly younger (mean 58.1 ± 14.8 years) than those in the cancer-related (64.2 ± 12.8 years) and non–cancer-related (68.8 ± 13.5 years) groups (*p* < 0.001). A significant difference in sex distribution was observed across groups (males: 33.6%, 50.5%, and 39.2%, respectively; *p* = 0.001).

Detailed demographic, oncologic, and pain-related characteristics are summarized in Table 1. The distribution of primary cancer types across groups is provided in Supplementary Table S1B, with breast cancer being the most common diagnosis.

**Table 1:** Baseline characteristics by pain etiology group. Baseline demographic, oncologic, and pain characteristics stratified by pain etiology: treatment-related pain (TRP), cancer-related pain not attributed to treatment, and non–cancer-related pain

Characteristic	Treatment-related pain (*n* = 262)	Cancer-related pain (non-treatment) (*n* = 194)	Non–cancer-related pain (*n* = 166)	*P* value
Demographics				
Age, mean ± SD	58.13 ± 14.83	64.24 ± 12.82	68.80 ± 13.49	< 0.001
Sex				0.001
Male	88 (33.6%)	98 (50.5%)	65 (39.2%)	
Female	174 (66.4%)	96 (49.5%)	101 (60.8%)	
Disease status				< 0.001
Active disease	117 (44.7%)	185 (95.4%)	103 (62.0%)	
Non-active disease	145 (55.3%)	9 (4.6%)	63 (38.0%)	
Current treatment status				< 0.001
On active treatment	116 (44.3%)	133 (68.6%)	91 (54.8%)	
Not on active treatment	146 (55.7%)	61 (31.4%)	75 (45.2%)	
Pain characteristics				
Pain duration > 6 months				< 0.001
Yes	67 (25.6%)	112 (57.7%)	74 (44.6%)	
No	195 (74.4%)	82 (42.3%)	92 (55.4%)	
Pain mechanism				< 0.001
Nociceptive	45 (17.2%)	69 (35.6%)	66 (39.8%)	
Neuropathic	121 (46.2%)	55 (28.4%)	76 (45.8%)	
Mixed	94 (35.9%)	69 (35.6%)	20 (12.0%)	
Other	2 (0.8%)	1 (0.5%)	4 (2.4%)	
Vital status at data cut	—	—	—	< 0.001
Alive	203 (77.5%)	72 (37.1%)	129 (77.7%)	
Dead	43 (16.4%)	111 (57.2%)	29 (17.5%)	
Missing	16 (6.1%)	11 (5.7%)	8 (4.8%)	
Among decedents: time from index visit to death	—	—	—	0.187
< 6 months	17 (39.5%)	49 (44.1%)	6 (20.7%)	
6–12 months	5 (11.6%)	19 (17.1%)	6 (20.7%)	
> 12 months	15 (34.9%)	34 (30.6%)	14 (48.3%)	
Missing	6 (14.0%)	9 (8.1%)	3 (10.3%)	

Values are mean ± SD or n (%). *P* values are from one-way ANOVA for continuous variables and chi-square tests for categorical variables. Etiology groups were defined using chart-coded variables: TRP (2end to Treatment = 1); cancer-related non-treatment pain (2end to Treatment = 2 and Pain 2end to Onco = 1); non–cancer-related pain (2end to Treatment = 2 and Pain 2end to Onco = 2). Vital status and time-to-death interval among decedents (< 6, 6–12, > 12 months; missing reported) are reported; missingness did not differ across groups (both *p* > 0.5)

*TRP* treatment-related pain, *SD* standard deviation, *ANOVA* analysis of variance

Patterns of missing data and the final analytic sample sizes for each analysis are provided in Supplementary Tables S4–S5.

Vital status at the time of data cutoff differed significantly across groups (*p* < 0.001), reflecting the underlying oncologic prognosis of each cohort. Among those who were deceased, the distribution of time-to-death intervals from the index clinic visit (< 6 months, 6–12 months, and > 12 months) did not differ significantly across groups (*p* = 0.187). Missing data patterns for vital status and for time-to-death interval among decedents were balanced across groups (both *p* > 0.5; Table 1).

### Factors associated with TRP attribution

In the primary multivariable model (excluding treatment-exposure variables), peripheral neuropathy was the strongest factor associated with TRP attribution (aOR 9.18; 95% CI 2.56–32.83; *p* < 0.001). Several factors were associated with significantly lower odds of TRP: pain duration > 6 months (aOR 0.31; 95% CI 0.20–0.50; *p* < 0.001), a nociceptive pain mechanism versus mixed (aOR 0.53; 95% CI 0.30–0.94; *p* = 0.029), and older age (aOR 0.977 per year; 95% CI 0.960–0.994; *p* = 0.007). A neuropathic pain mechanism (vs mixed) was associated with higher odds of TRP attribution (aOR 1.85; 95% CI 1.07–3.21; *p* = 0.029).

**Fig. 1:**
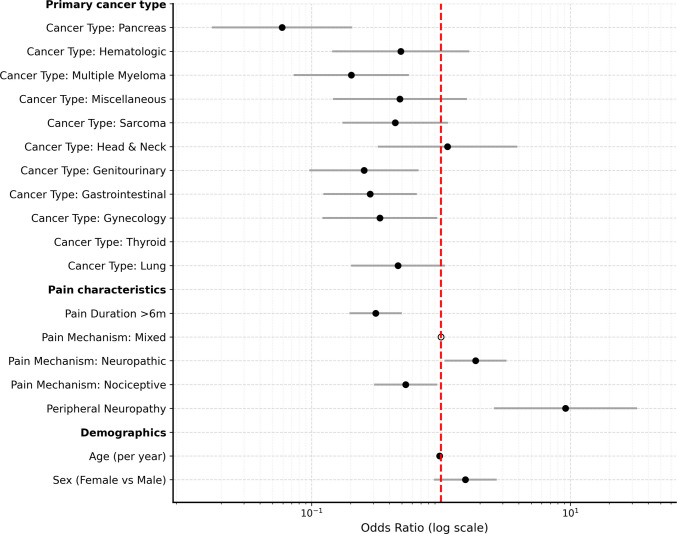
Independent clinical correlates of treatment-related pain

These findings are visualized in Fig. 1 and detailed in Supplementary Table S3A. Specific primary cancer types—including pancreatic, multiple myeloma, gastrointestinal, genitourinary, and OBGYN malignancies—demonstrated lower odds of TRP compared with breast cancer (all *p* < 0.05). Sensitivity analyses incorporating cancer-directed treatment exposures (Supplementary Table S3B) did not materially alter the primary model’s overall pattern of associations.

### Pain mechanism by etiology

**Fig. 2:**
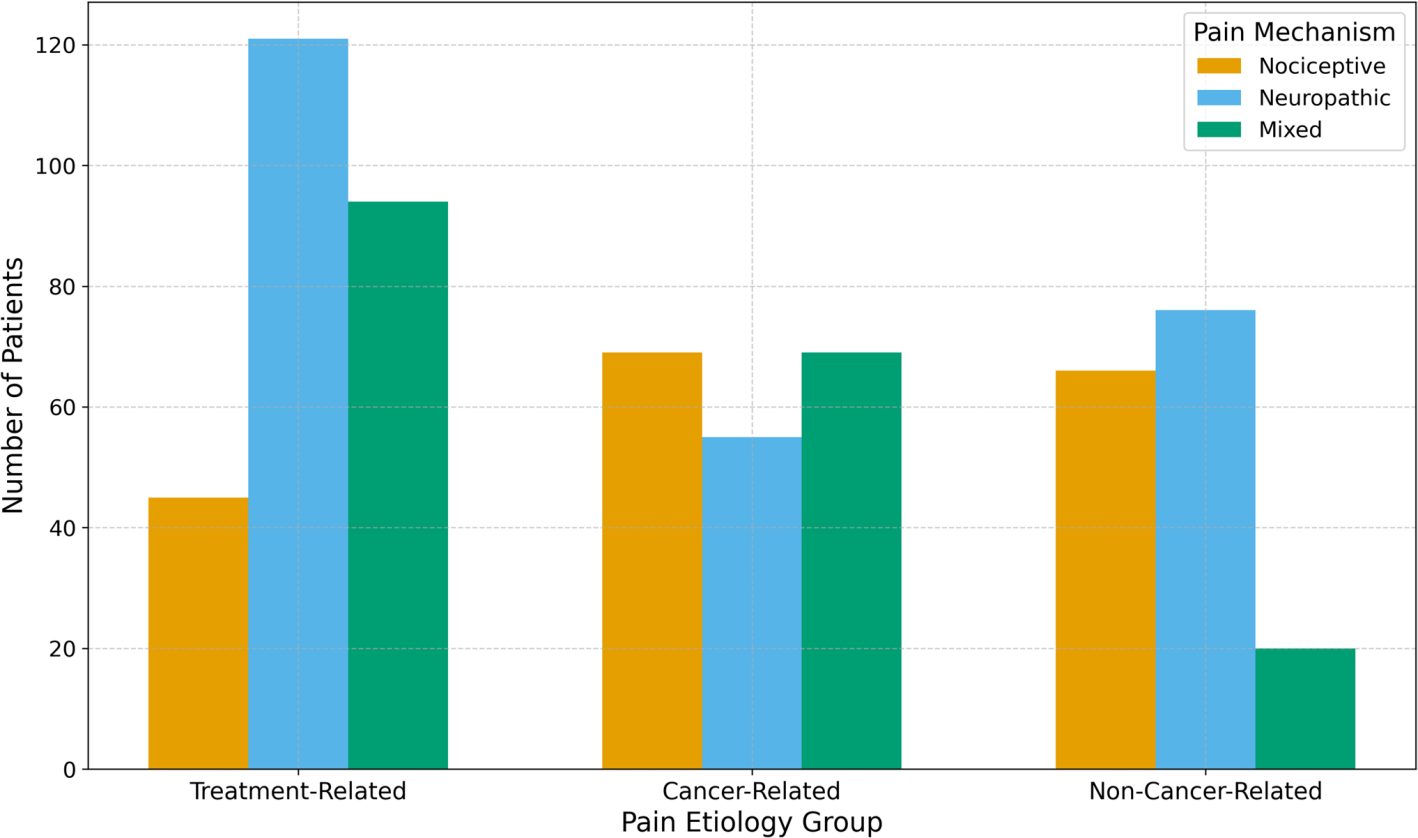
Pain mechanism distribution by pain etiology subgroup

After the exclusion of seven patients classified as having “Other” mechanisms, 615 patients remained for analysis. The distribution of nociceptive, neuropathic, and mixed mechanisms differed significantly across etiology groups (χ^2^ = 55.9, df = 4, *p* < 0.001; Cramér’s V = 0.213). Neuropathic pain was the most prevalent mechanism within the TRP group (46.2%), whereas nociceptive and mixed mechanisms were similarly distributed among the tumor-related group, and mixed pain was relatively uncommon in the cancer-unrelated group. Comprehensive mechanism counts and statistics are reported in Supplementary Table S1 and visualized in Fig. 2.

### Pain-diagnosis patterns

**Fig. 3:**
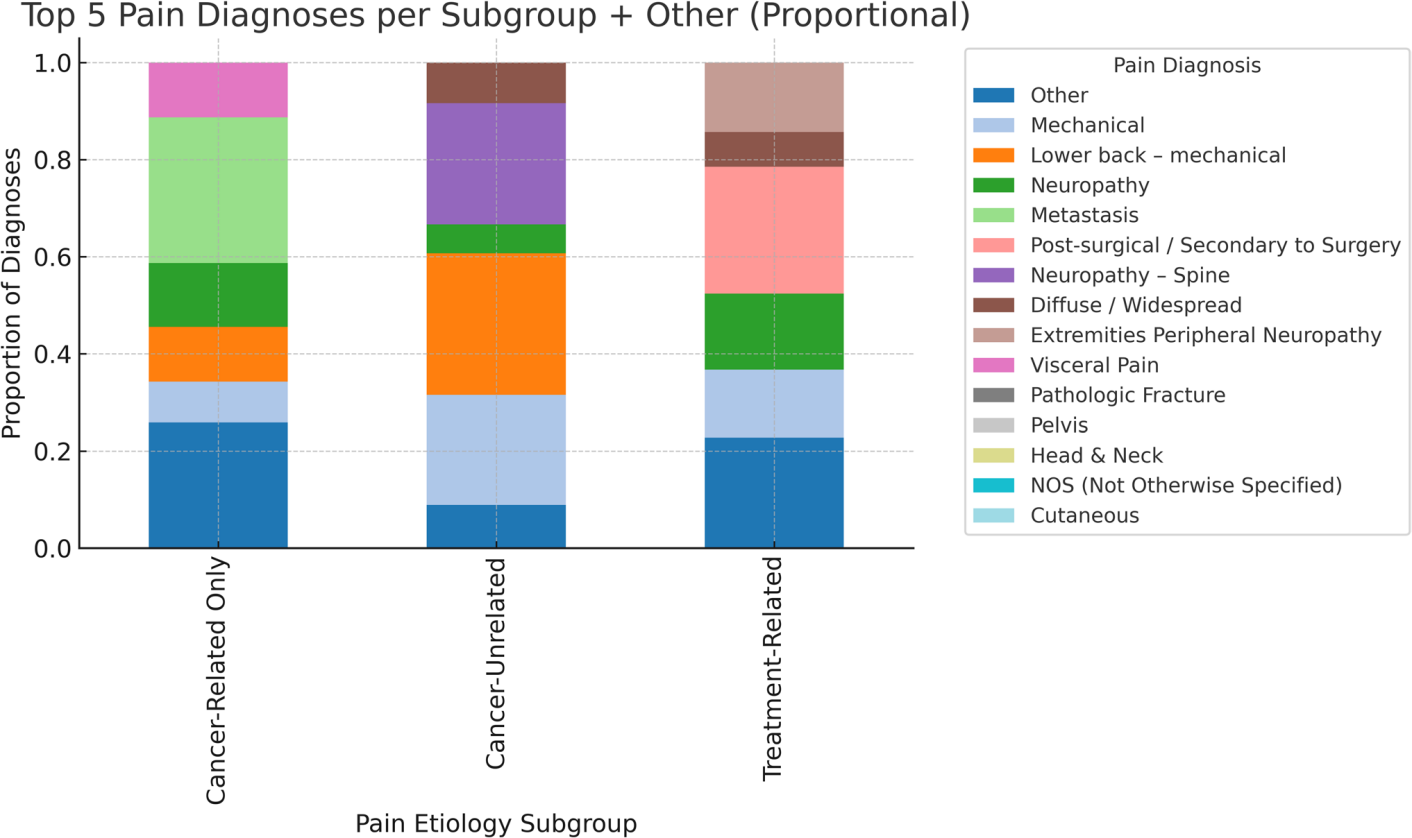
Distribution of top pain diagnoses by etiological subgroup

Diagnostic profiles revealed distinct patterns by etiology. TRP patients were most frequently diagnosed with peripheral neuropathy and post-surgical pain syndromes. In contrast, metastasis-related and visceral pain were predominant among cancer-related (non-treatment) cases. The non–cancer-related group demonstrated a heterogeneous range of chronic pain syndromes, primarily mechanical and degenerative conditions. The top diagnostic categories for each group are displayed in Fig. 3, with the complete diagnostic distribution provided in Supplementary Table S2.

### Interventional procedures and cannabis use

Interventional procedure offering and completion differed across groups. Despite higher rates of neuropathic and mixed pain mechanisms, TRP patients were less likely to undergo interventional procedures.

Procedures were offered to 44.7% of TRP patients versus 49.2% of those with non–cancer-related pain and were successfully completed in 30.9% versus 37.5%, respectively. This corresponds to an absolute difference of 6.6 percentage points and a 17.6% relative reduction in completion for the TRP cohort (relative to the non–cancer-related group). Medical cannabis prescription rates remained similar across all groups, as detailed in Table 1.

## Discussion

This study demonstrates that treatment-related pain (TRP) constitutes a substantial component of refractory cancer pain in a tertiary referral setting, accounting for over 40% of cases and highlighting its central role in this population. In contrast to tumor-related pain, TRP was more common among younger patients and those with more favorable survival, supporting its characterization as a survivorship-associated morbidity rather than a phenomenon confined to advanced disease. These findings suggest that persistent pain in cancer populations is increasingly driven by the long-term consequences of cancer treatment, with direct implications for supportive care delivery.

While individual treatment-related pain syndromes are well described, this study provides a comprehensive characterization within a refractory tertiary cohort, highlighting their cumulative burden and implications for supportive care services.

TRP patients were younger (mean 58.1 years) and demonstrated the highest survival (77.5%) compared with those with active malignancy. In multivariable analysis, increasing age was associated with lower odds of TRP versus tumor-related pain (aOR 0.977 per year; 95% CI 0.960–0.994). Overall, these findings indicate that TRP is concentrated among patients with more favorable oncologic trajectories, who may live longer with refractory pain, although lifetime pain burden was not assessed. The combination of younger age and higher survivorship underscores TRP as a prominent survivorship morbidity that warrants dedicated recognition and mechanism-aligned management, rather than being framed primarily within end-of-life care.

Given that all patients were referred for refractory pain following inadequate response to standard pharmacologic management, a substantial functional burden is likely, even though detailed functional outcomes were not systematically captured. This is particularly relevant in patients with otherwise stable disease, including cancer survivors under oncologic surveillance without active disease, in whom persistent pain may represent a dominant determinant of functional impairment.

These findings have direct implications for supportive care pathways. Patients with treatment-related pain often present with complex neuropathic and mixed phenotypes that may not respond adequately to conventional opioid-centered approaches. In routine oncology practice, such symptoms may be under-recognized or misattributed to disease progression, potentially delaying mechanism-based interventions. This misattribution may also lead to escalation of conventional analgesics without addressing the underlying mechanism. The high prevalence of TRP in this refractory cohort supports earlier integration of multidisciplinary supportive care, including pain specialists, rehabilitation, and interventional strategies tailored to pain phenotype.

The high prevalence of TRP (42.1%) likely reflects our tertiary referral setting, which preferentially captures patients with refractory syndromes such as chemotherapy-induced peripheral neuropathy (CIPN), post-radiation fibrosis, and persistent post-surgical pain. These conditions may respond incompletely to opioid-centered regimens and often require a broader plan beyond medication alone, including rehabilitation and targeted procedures. In selected cases, management in a specialized pain clinic may include peripheral nerve blocks or soft-tissue plane injections, infusion-based therapies such as intravenous lidocaine, and neuromodulation when clinically appropriate, based on the pain phenotype and functional priorities.

Although chronic pain is typically defined as ≥ 3 months (IASP/ICD-11), we used a ≥ 6-month threshold to focus on more persistent pain in this refractory cohort. This operational choice, together with the referral-based design, may influence attribution patterns and should be interpreted in the context of a tertiary referral population rather than as a true time-dependent shift in mechanism. This may also suggest that treatment-related pain is more readily recognized earlier in the course of persistent symptoms, whereas later pain is more often attributed to disease progression or recurrence.

TRP was characterized by substantial mechanistic complexity, with neuropathic pain (46.2%) and mixed mechanisms (35.9%) most common. Unlike tumor-related pain, TRP often does not have a discrete space-occupying lesion on routine imaging (although fibrosis or postsurgical change may contribute), and imaging that shows no progression may still miss processes such as small-fiber neurotoxicity or central sensitization. Consistent with this, the non-cancer-related pain group had a much lower rate of mixed pain (12.3%) than the TRP group (~ 36%). Diagnostic patterns also differed: metastatic and visceral pain predominated in tumor-related pain, whereas peripheral neuropathy and post-surgical pain were more characteristic of TRP. Together, these findings suggest that TRP assessment should rely less on imaging alone and more on careful history-taking and focused physical examination, integrating the temporal and anatomic relationship between cancer treatment and the pain phenotype.

In secondary analyses, TRP differed from other groups in interventional procedure completion, while medical cannabis use rates were similar across groups. Because these variables were not pre-specified outcomes and TRP-specific comparative data are limited, we interpret these findings cautiously and refrain from causal inference. Nonetheless, these preliminary observations may help motivate future prospective studies examining the role and timing of interventional approaches and nontraditional pharmacologic strategies in TRP and whether their use is aligned with the dominant pain mechanism.

## Limitations

While this study offers a unique characterization of TRP, several limitations should be considered. First, the single-center, tertiary-clinic design introduces selection bias, as our cohort represents patients referred for refractory pain following inadequate response to standard pharmacologic management. Therefore, the prevalence and mechanistic complexity of TRP should be interpreted with caution when generalizing to the broader survivor population.

Second, the retrospective design relies on the quality of clinical documentation. Although we used a structured multi-physician coding framework, some granular treatment-exposure details (e.g., radiotherapy dosages) were unavailable. In addition, we could not determine whether differences in interventional procedure completion were driven by clinician- or patient-related factors.

We did not systematically capture detailed functional outcomes, such as work capacity or patient-reported interference with daily activities. Although ECOG performance status was available, it may not fully reflect pain-related functional impairment. Given the refractory nature of the cohort, a substantial functional burden is likely; however, this could not be formally quantified. Future studies should incorporate standardized measures of functional impact to better define the clinical burden of treatment-related pain.

Finally, pain mechanisms were determined by expert clinical evaluation rather than quantitative sensory testing (QST), which may have provided a more standardized physiologic characterization.

## Conclusion

Treatment-related pain (TRP) is a prevalent and under-recognized consequence of improved cancer survival. In this tertiary registry, TRP was more common among younger survivors and was characterized predominantly by neuropathic and mixed pain phenotypes. These findings position TRP as a key survivorship morbidity and support the need for survivorship-focused, mechanism-informed assessment pathways. Earlier recognition and integration of multidisciplinary supportive care strategies may improve outcomes for patients with persistent treatment-related pain.

## Supplementary Information

Below is the link to the electronic supplementary material.

ESM 1 (DOCX 15.6 KB)

ESM 2 (XLSX 10.0 KB)

ESM 3 (DOCX 14.0 KB)

ESM 4 (DOCX 27.1 KB)

ESM 5 (XLSX 9.91 KB)

ESM 6 (XLSX 10.6 KB)

ESM 7 (DOCX 13.8 KB)

ESM 8 (XLSX 11.0 KB)

ESM 9 (DOCX 13.9 KB)

ESM 10 (XLSX 10.6 KB)

ESM 11 (DOCX 13.6 KB)

ESM 12 (XLSX 8.97 KB)

ESM 13 (DOCX 28.0 KB)

## Data Availability

The datasets generated during and/or analyzed during the current study are not publicly available due to patient privacy and clinical confidentiality but are available from the corresponding author on reasonable request.
