# Supplementary material for: Treatment-related pain in refractory cancer pain: prevalence, mechanisms, and clinical implications in a tertiary referral cohort
Source: Support Care Cancer. 2026 Jun 12;34(7):647. doi: 10.1007/s00520-026-10886-6 (PMC13260140; doi:10.1007/s00520-026-10886-6)
Supplement: Supplementary file 1 — (DOCX 15.6 KB) [file 520_2026_10886_MOESM1_ESM.docx]

# Pain Mechanism Distribution by Pain Etiology Subgroup

| Pain Mechanism | Treatment-Related (n=260) | Cancer-Related Only (n=193) | Cancer-Unrelated (n=162) |
| --- | --- | --- | --- |
| Nociceptive | 45 | 69 | 66 |
| Neuropathic | 121 | 55 | 76 |
| Mixed | 94 | 69 | 20 |

# Caption: Distribution of pain mechanisms (nociceptive, neuropathic, and mixed) across three clinically defined pain etiology subgroups: treatment-related pain (TRP), cancer-related pain only, and cancer-unrelated pain.

# Abbreviations: TRP – Treatment-Related Pain.

# Footnotes:

# Patients with “Other” pain types (n = 7) were excluded from this analysis.

# Mixed pain refers to overlapping nociceptive and neuropathic mechanisms as defined by clinical documentation.

# Subgroups were mutually exclusive, defined using structured coding (see Methods).
