# Supplementary material for: Treatment-related pain in refractory cancer pain: prevalence, mechanisms, and clinical implications in a tertiary referral cohort
Source: Support Care Cancer. 2026 Jun 12;34(7):647. doi: 10.1007/s00520-026-10886-6 (PMC13260140; doi:10.1007/s00520-026-10886-6)
Supplement: Supplementary file 3 — (DOCX 14.0 KB) [file 520_2026_10886_MOESM3_ESM.docx]

**Supplementary Table S2. Full Pain Diagnosis Distributions by Pain Etiology Group**

This table displays the absolute counts and proportions of pain diagnoses across three mutually exclusive pain etiology groups: treatment-related pain (TRP), cancer-related pain (non-treatment), and cancer-unrelated pain. Diagnoses were assigned based on structured clinical documentation at the time of registry enrollment. Peripheral neuropathy and post-surgical pain were the most common diagnoses in the TRP group. Metastatic and visceral pain predominated in the cancer-related group, while the cancer-unrelated group exhibited a diverse profile, including mechanical and chronic non-malignant pain syndromes.

**Footnote**:
Diagnoses were recorded based on structured clinician documentation at registry enrollment. Patients may have more than one diagnosis. Percentages reflect the proportion of total diagnoses recorded within each etiology subgroup (column-wise). Diagnoses with very low frequency were included for completeness.

**Abbreviations**:
TRP = Treatment-Related Pain;
MSK = Musculoskeletal;
GI = Gastrointestinal;
CNS = Central Nervous System.
