# Supplementary material for: Treatment-related pain in refractory cancer pain: prevalence, mechanisms, and clinical implications in a tertiary referral cohort
Source: Support Care Cancer. 2026 Jun 12;34(7):647. doi: 10.1007/s00520-026-10886-6 (PMC13260140; doi:10.1007/s00520-026-10886-6)
Supplement: Supplementary file 4 — (DOCX 27.1 KB) [file 520_2026_10886_MOESM4_ESM.docx]

# Supplementary Table S1B. Distribution of Primary Cancer Types by Pain Etiology Group

# Description: This table illustrates the distribution of primary oncological diagnoses across three distinct pain etiology groups. The data provides a breakdown of how the source of pain (etiology) varies among different cancer types within the study population.

# Pain Etiology Definitions:

# Treatment-Related Pain: Pain directly resulting from oncological interventions, including surgical procedures, chemotherapy-induced toxicities (e.g., peripheral neuropathy), or radiation-induced injuries.

# Cancer-Related Pain: Pain caused directly by the malignancy, such as tumor infiltration into tissues, bone metastases, or nerve compression by the primary tumor.

# Non-Cancer Related / Mixed: Pain attributed to mechanisms unrelated to the oncologic diagnosis (e.g., pre-existing chronic pain, osteoarthritis) or cases involving multifactorial/combined mechanisms where a single primary source could not be isolated.

# Table Legend & Notes:

# Data Format: Values are presented as n, representing the frequency of patients within each cancer category.

# Population Note: While the total study population consists of 622 patients, the total number of diagnoses in this table is 633. This discrepancy is due to a subset of patients presenting with multiple primary malignancies or overlapping cancer types.
