# Supplementary material for: Treatment-related pain in refractory cancer pain: prevalence, mechanisms, and clinical implications in a tertiary referral cohort
Source: Support Care Cancer. 2026 Jun 12;34(7):647. doi: 10.1007/s00520-026-10886-6 (PMC13260140; doi:10.1007/s00520-026-10886-6)
Supplement: Supplementary file 7 — (DOCX 13.8 KB) [file 520_2026_10886_MOESM7_ESM.docx]

**Supplementary Table S3A footnote**

Multivariable logistic regression with treatment-related pain (TRP) as the binary outcome (yes/no). The comparison group comprised patients with tumor-related pain (cancer-related non-treatment pain only). The primary model excluded cancer-directed treatment exposure variables (surgery, radiotherapy, systemic therapy) to reduce incorporation bias. Pain mechanism was entered as an indicator-coded categorical variable with mixed pain as the reference category (nociceptive vs mixed; neuropathic vs mixed). Reference categories: male sex; pain duration ≤6 months; no peripheral neuropathy; breast cancer (primary cancer type). The heterogeneous “Other” pain mechanism category was excluded; complete-case analytic N=453. Results are reported as adjusted odds ratios (aORs) with 95% confidence intervals.

**Supplementary Table S3B footnote**

Model specification as in Table S3A, with additional covariates for prior cancer-directed treatment exposures (surgery, radiotherapy, and systemic therapy). Pain mechanism reference category was mixed pain (nociceptive vs mixed; neuropathic vs mixed). Reference categories: male sex; pain duration ≤6 months; no peripheral neuropathy; no exposure to each treatment modality (for treatment-exposure variables); breast cancer (primary cancer type). The heterogeneous “Other” pain mechanism category was excluded; complete-case analytic N=453. Results are reported as adjusted odds ratios (aORs) with 95% confidence intervals.
