# Supplementary material for: Treatment-related pain in refractory cancer pain: prevalence, mechanisms, and clinical implications in a tertiary referral cohort
Source: Support Care Cancer. 2026 Jun 12;34(7):647. doi: 10.1007/s00520-026-10886-6 (PMC13260140; doi:10.1007/s00520-026-10886-6)
Supplement: Supplementary file 9 — (DOCX 13.9 KB) [file 520_2026_10886_MOESM9_ESM.docx]

**Supplement Table S4. Missingness by variable (N=622)**

**Caption:**
Variable-level missingness for demographics, oncologic variables, pain characteristics, treatment exposures, survival variables, and utilization outcomes used in descriptive and multivariable analyses.

**Footnote:**

Missing data are reported as count (%) of the full cohort (N=622). “Time-to-death category” is defined only among decedents and is therefore expected to be missing for patients who were alive at last follow-up. Logistic regression analyses were conducted using complete cases for all model covariates, restricted to TRP and tumor-related pain, after excluding the heterogeneous “Other” pain mechanism category (analytic N=453). Survival analyses were restricted to patients with known survival status (analytic N=587).

**Abbreviations:**
TRP, treatment-related pain.
