# Supplementary material for: Treatment-related pain in refractory cancer pain: prevalence, mechanisms, and clinical implications in a tertiary referral cohort
Source: Support Care Cancer. 2026 Jun 12;34(7):647. doi: 10.1007/s00520-026-10886-6 (PMC13260140; doi:10.1007/s00520-026-10886-6)
Supplement: Supplementary file 11 — (DOCX 13.6 KB) [file 520_2026_10886_MOESM11_ESM.docx]

**Supplement Table S5. Study flow by analysis set (N=622)**
**Caption:**
Derivation of analytic sets for descriptive analyses, regression models, pain mechanism analyses, and survival analyses.

**Footnote:**
Etiology-based analyses and Table 1 include all patients with assigned pain etiology (N=622). Analyses involving pain mechanism excluded the heterogeneous “Other” pain mechanism category (n=7), yielding N=615. Regression analyses were complete-case for all model covariates among eligible participants restricted to TRP and tumor-related pain (N=453). Survival analyses were restricted to patients with known survival status (N=587).

**Abbreviations:**
TRP, treatment-related pain.
