# Supplementary material for: Treatment-related pain in refractory cancer pain: prevalence, mechanisms, and clinical implications in a tertiary referral cohort
Source: Support Care Cancer. 2026 Jun 12;34(7):647. doi: 10.1007/s00520-026-10886-6 (PMC13260140; doi:10.1007/s00520-026-10886-6)
Supplement: Supplementary file 13 — (DOCX 28.0 KB) [file 520_2026_10886_MOESM13_ESM.docx]

# eAppendix. Variable definitions and coding rules

This appendix summarizes operational definitions and coding rules applied to variables extracted from the refractory cancer pain clinic registry and source electronic medical records.

## Pain etiology groups

Treatment-related pain (TRP): Pain attributed to cancer-directed treatment (systemic therapy, surgery, or radiotherapy) based on documented syndrome pattern, timing, and anatomic distribution compatible with the exposure.

Cancer-related pain, non-treatment: Pain attributed to malignancy (e.g., tumor burden, metastasis, visceral involvement) without sufficient documentation to support attribution to cancer-directed therapy.

Non–cancer-related pain: Pain not attributed to cancer or its treatment based on clinical documentation (e.g., degenerative spine disease, osteoarthritis, primary headache disorders, chronic non-malignant pain syndromes).

If documentation was insufficient to assign etiology with confidence, the case was excluded from etiology-based analyses.

## TRP attribution framework (syndrome- and timing-compatible)

TRP was operationalized using predefined syndrome patterns with timing and distribution compatible with treatment exposure. Examples included:

- Systemic therapy–associated syndromes (e.g., chemotherapy-induced peripheral neuropathy).
- Persistent post-surgical pain syndromes, including neuropathic pain localized to the surgical field with compatible sensory features.
- Radiotherapy-associated syndromes (e.g., plexopathy or fibrosis-related pain) with anatomic concordance to the treated region.

## Pain mechanism classification

Pain mechanism was coded by clinicians as nociceptive, neuropathic, mixed, or other based on the index clinic evaluation.

Nociceptive pain was defined as musculoskeletal, soft-tissue, or visceral pain without neuropathic descriptors; neuropathic pain followed the updated grading framework (definite/probable/possible); mixed pain was assigned when nociceptive and neuropathic features co-occurred at the same site.

When multiple pain mechanisms were present, a dominant mechanism was assigned based on the primary driver of the chief pain complaint at the index evaluation (as documented by the clinician). Infrequent or heterogeneous presentations were coded as “Other” and excluded from regression models due to small numbers (n=7).

## Key covariates used in regression models

Age: Recorded in years at index clinic visit; modeled per 1-year increase.

Sex: Female vs male (male reference).

Pain duration >6 months: Binary indicator of pain duration at index evaluation (>6 months vs ≤6 months).

Peripheral neuropathy: Binary indicator derived from clinician-coded diagnosis of peripheral neuropathy in the registry.

Primary cancer type: Categorized from registry diagnosis fields; modeled as indicator variables with breast cancer as the reference category.

Cancer-directed treatment exposures (sensitivity model): Binary indicators for prior systemic therapy, radiotherapy, and surgery exposures (included only in the sensitivity model).

Pain mechanism: Entered as indicator variables for neuropathic and nociceptive, with mixed as the reference category.

## Handling of missingness and analytic sets

Variable-level missingness is summarized in Supplement Table Sx. Logistic regression analyses were conducted using complete cases for all model covariates, restricted to TRP and tumor-related pain, after excluding the “Other” pain mechanism category (final N=453). Survival analyses were restricted to patients with known survival status (final N=587).

## Inter-rater reliability

Inter-rater reliability statistics (e.g., kappa) were not computed. Instead, the workflow incorporated multi-physician abstraction, structured coding fields, secondary quality control, and return-to-chart verification for discrepant or ambiguous entries.
